# Brown Adipose Tissue Thermogenic Capacity Is Regulated by Elovl6

**DOI:** 10.1016/j.celrep.2015.11.004

**Published:** 2015-11-25

**Authors:** Chong Yew Tan, Samuel Virtue, Guillaume Bidault, Martin Dale, Rachel Hagen, Julian L. Griffin, Antonio Vidal-Puig

**Affiliations:** 1 University of Cambridge Metabolic Research Laboratories, Wellcome Trust-MRC Institute of Metabolic Science, Addenbrooke’s Hospital, Cambridge CB2 0QQ, UK; 2 Medical Research Council Human Nutrition Research, Cambridge CB1 9NL, UK; 3 The Department of Biochemistry and the Cambridge Systems Biology Centre, Tennis Court Road, Cambridge CB2 1GA, UK; 4 Wellcome Trust Sanger Institute, Wellcome Trust Genome Campus, Hinxton, Cambridgeshire CB10 1SA, UK

**Keywords:** elongase, Elovl6, brown adipose tissue, adaptive thermogenesis, fatty acid acyl chain length, energy expenditure

## Abstract

Although many transcriptional pathways regulating BAT have been identified, the role of lipid biosynthetic enzymes in thermogenesis has been less investigated. Whereas cold exposure causes changes in the fatty acid composition of BAT, the functional consequences of this remains relatively unexplored. In this study, we demonstrate that the enzyme Elongation of Very Long Chain fatty acids 6 (Elovl6) is necessary for the thermogenic action of BAT. Elovl6 is responsible for converting C16 non-essential fatty acids into C18 species. Loss of Elovl6 does not modulate traditional BAT markers; instead, it causes reduced expression of mitochondrial electron transport chain components and lower BAT thermogenic capacity. The reduction in BAT activity appears to be counteracted by increased beiging of scWAT. When beige fat is disabled by thermoneutrality or aging, Elovl6 KO mice gain weight and have increased scWAT mass and impaired carbohydrate metabolism. Overall, our study suggests fatty acid chain length is important for BAT function.

## Highlights

• The fatty acid elongase Elovl6 is a thermogenically regulated gene in BAT
• Elovl6 is necessary for full thermogenic recruitment of brown adipose tissue
• Elovl6 acts by regulating mitochondrial function in brown adipose tissue

## Graphical Abstract

**Figure:**
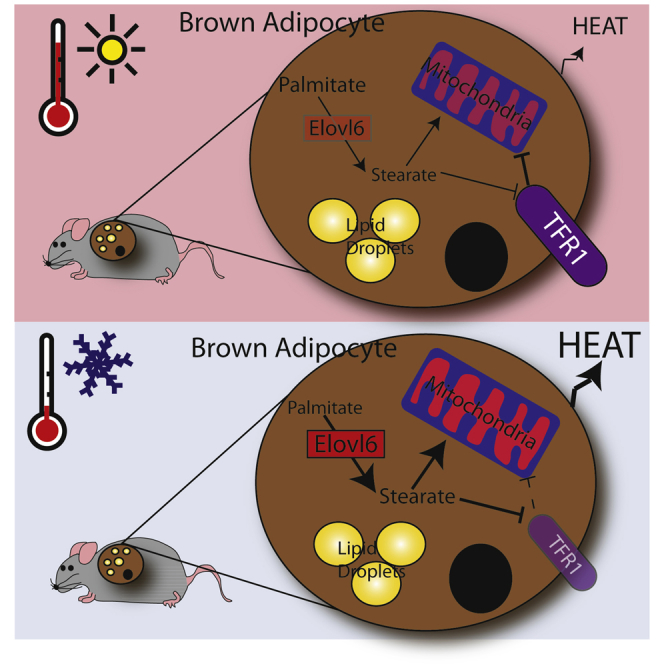

## Teaser

Tan et al. find that the elongation of non-essential C16 fatty acids to C18 species regulates mitochondrial function and is necessary for full recruitment of heat-generating brown adipose tissue.

## Introduction

Brown adipose tissue (BAT) is responsible for heat generation, a process that occurs due to the uncoupling of electron transport from ATP synthesis by uncoupling protein 1 (UCP1). The most potent environmental factor regulating BAT is temperature. In response to an acute reduction in environmental temperature (cold exposure), BAT is immediately activated to produce heat. If an animal is maintained at a lower environmental temperature, BAT is recruited, thus increasing the thermogenic capacity of the animal. When the amount of BAT present matches the requirements of the new temperature, then an animal is described as cold acclimated (Cannon and Nedergaard, 2004). A switch between room temperature (24°C) and cold (5°C) requires 4 weeks for full cold acclimation to occur in mice. The sympathetic nervous system (SNS) is, perhaps, the single most important factor controlling BAT activation and acclimation. In response to cold exposure, SNS tone to BAT increases causing both the activation of UCP1 and initiating a series of transcriptional changes that lead to BAT recruitment. While many transcriptional pathways regulating BAT recruitment have been identified, there has been less focus on the role of lipid metabolism within BAT, other than as a source of fuel (Bartelt et al., 2011).

Some previous studies have investigated changes in the lipidome of BAT in response to cold. Cold acclimation causes a remodeling of the BAT lipidome and in particular the mitochondrial lipidome (Ocloo et al., 2007). In addition to changes in the BAT lipidome, it has long been known that enzymes responsible for modifying fatty acid chain length are regulated in BAT in response to cold. Indeed, one of the most upregulated genes in BAT when comparing mice housed at 5°C to those housed at 30°C is the fatty acid elongase Elovl3 (formally known as Cold Inducible Gene 30) (Jakobsson et al., 2005, Westerberg et al., 2006). Elovl3 is responsible for the production of C20, C22, and C24 fatty acid from C18 precursors, and mice lacking Elovl3 showed defects in lipid recruitment to BAT in response to cold exposure (Westerberg et al., 2006). However, Elovl3 products make up less than 1% of the cellular lipidome of BAT, raising questions regarding the role of more common fatty acids in the process of cold adaptation.

The fatty acid elongase Elovl6 is also known to be highly expressed in BAT (Moon et al., 2001). Elovl6 acts to convert C16 saturated and monounsaturated fatty acids to C18 fatty acids and can potentially affect over 50% of the cellular lipidome. In addition, the Elovl6 product stearate has recently been implicated in the regulation of mitochondrial function via steroylation of the Transferrin 1 receptor (TfR1) (Senyilmaz et al., 2015). Given that BAT has one of the highest mitochondrial densities of any tissue, these data suggest that Elovl6 may act to regulate mitochondrial function and therefore thermogenesis in BAT.

Mice lacking Elovl6 have been previously investigated in terms of carbohydrate metabolism and hepatosteatosis, with discordant results being reported by two separate groups (Matsuzaka et al., 2007, Moon et al., 2014). In this present study, we identify Elovl6 as being thermogenically regulated and necessary for the adaptation of BAT to cold exposure.

## Results

**Figure 1:**
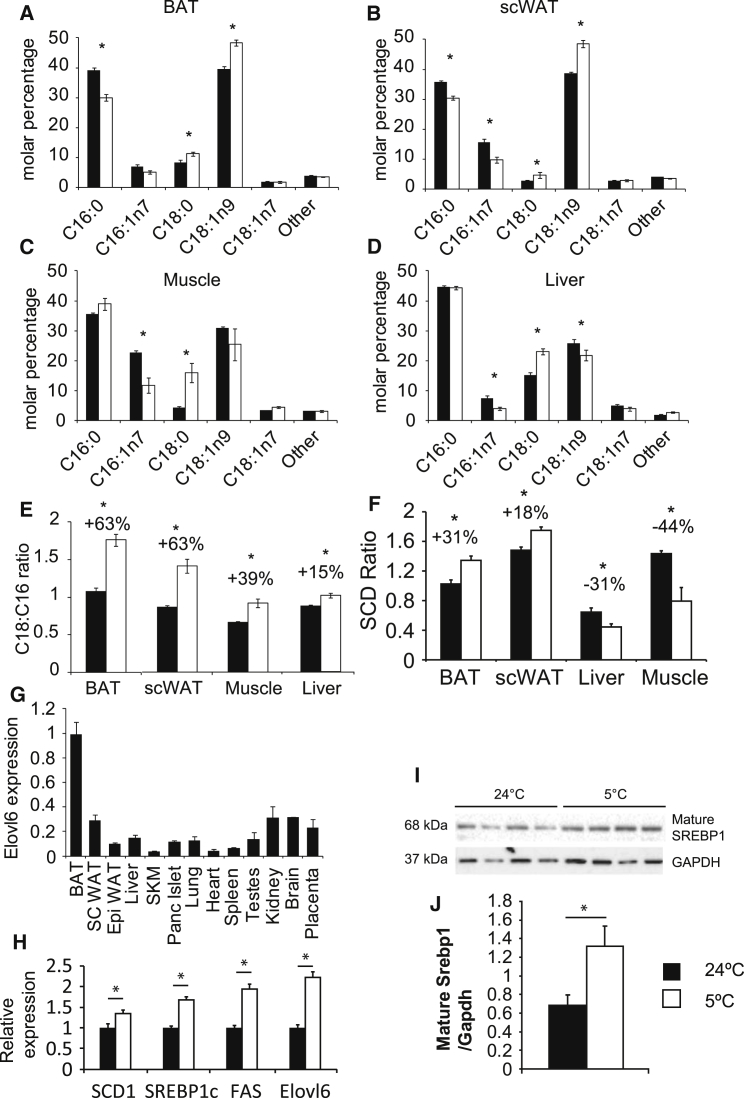
FAME Analysis of C16 and C18 Non-essential Fatty Acids of Tissues from Wild-Type Mice Acclimatized to Either 24°C or 5°C (A) BAT. (B) Inguinal Subcutaneous WAT. (C) Muscle. (D) Liver. (E) C18:C16 ratios for tissues. (F) SCD ratios for tissues. (G) Tissue distribution of Elovl6 expression. (H) Expression of Elovl6 and other DNL genes in BAT from mice housed at either 5°C or 24°C. (I) Western blot for nuclear SREBP1 from BAT from mice housed at either 5°C or 24°C. (J) Quantification of nSREBP. For FAME, n = 8 mice per group for BAT, scWAT, and liver at both temperatures and muscle at 24°C, n = 5 mice per group for muscle at 5°C; n = 8 mice per group for tissue distribution and Elovl6 expression, C57Bl/6J male 3–4 months of age. For western blotting, n = 4 per group. All groups compared by t test. Error bars ± SEM. ^∗^p < 0.05. Linked to Figure S1.

We initially investigated the changes in fatty acid chain length in BAT and other metabolically relevant organs in response to cold acclimation. Our results indicated that the most consistent and substantive changes were a shift toward C18 from C16 non-essential fatty acids in response to cold (Figures 1A–1D). In order to summarize these changes, we generated the C18:C16 ratio (see Figure S1A for equation), which represented elongation and the SCD ratio to summarize desaturation by the SCD class of enzymes (see Figure S1A for equation).

It was notable that the C18:C16 ratio, in response to cold, was most strongly increased in BAT and subcutaneous WAT; a major site of beige cell recruitment (Figure 1E). Conversely, in response to cold, the SCD ratio was induced to a smaller degree than the C18:C16 ratio in BAT and scWAT and actually decreased in liver and muscle (Figure 1F). The main enzyme responsible for elongation of C16 to C18 is thought to be Elovl6 (Moon et al., 2001, Ohno et al., 2010). We measured the expression profile of Elovl6 across multiple tissues at room temperature and determined that, consistent with previous reports (Moon et al., 2001), the expression of Elovl6 was highest in BAT, followed by inguinal subcutaneous WAT (scWAT), liver, and finally muscle, a sequence that mirrored the respective C18:C16 ratios of the tissues (Figure 1G). Additionally, Elovl6 expression was induced by cold acclimation in BAT (Figure 1H), consistent with the increased C18:C16 ratio of this tissue. In addition to increased expression of Elovl6 in the cold, *Fasn* and *Scd1* were also induced (Figure 1H). As *Elovl6* and *Fasn* are known SREBP1 targets, we measured the levels of the active nuclear form of SREBP1, which was increased by cold exposure (Figures 1I and 1J). To confirm the regulation of Elovl6 by SREBP1 in BAT, we measured expression of *Elovl6* and *Fasn* in BAT from *Srebp1c* KO mice and determined that Elovl6 expression was reduced (Figure S1B). Overall, our data suggested that cold acclimation caused a shift toward an increased C18 FA proportion, and that Elovl6 was a likely candidate to mediate this process.

To investigate the role of Elovl6 further, we utilized the ELOVL6 KO mouse. Elovl6 KO mice had previously been reported to be born at sub-Mendelian ratios (30% survival of KO mice) due to embryonic/early post-natal lethality (Matsuzaka et al., 2007, Moon et al., 2014). When bred at 21°C, Elovl6 KO mice exhibited a 30% survival rate (χ2 p = 0.0003, n = 84); however, when housed at 24°C the Elovl6 KO mice had a normal Mendelian ratio (χ2 p = 0.110 n = 120). This result was reminiscent of the low early post-natal survival rates of other mouse models with impaired thermogenic function (Adams et al., 2008).

**Figure 2:**
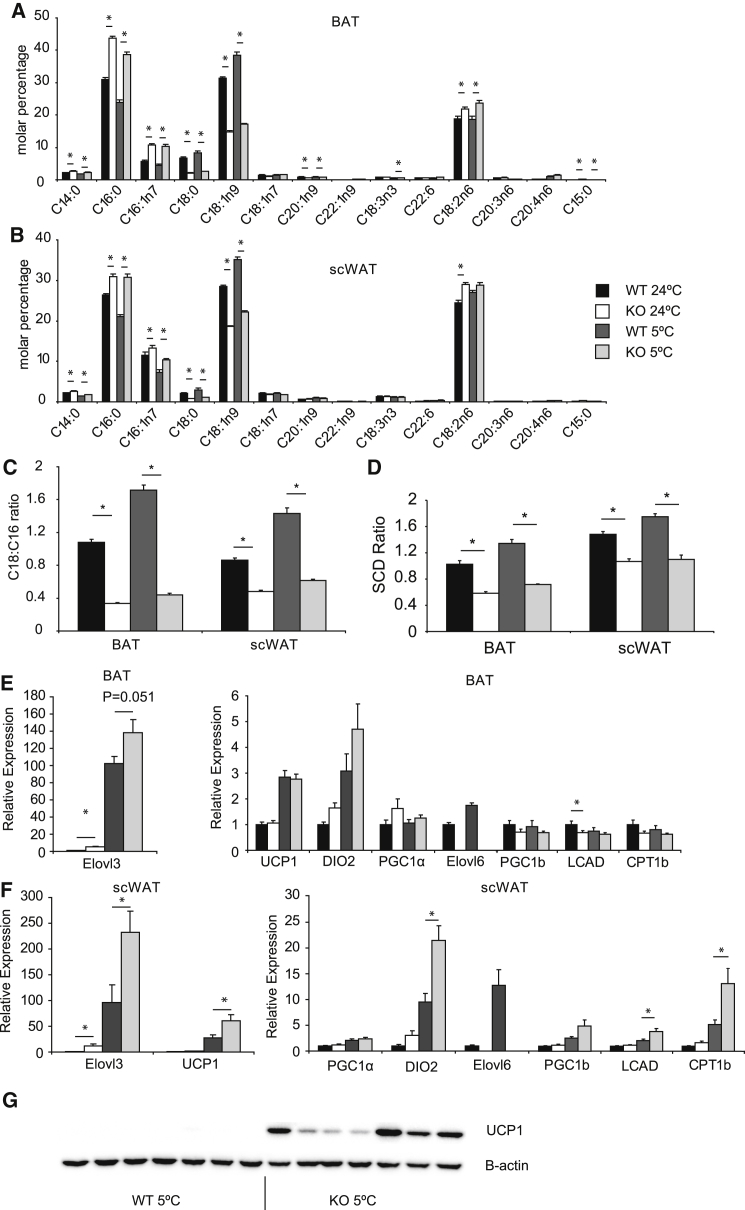
Fatty Acid Methyl Ester Analysis of Tissues from Wild-Type or Elovl6 KO Mice Acclimatized to Either 24°C or 5°C (A) BAT. (B) scWAT. (C) C18:C16 ratios for BAT and scWAT. (D) SCD ratios for BAT and scWAT. (E) Gene expression from BAT of WT and Elovl6 KO mice acclimated to either 24°C or 5°C. (F) Gene expression from inguinal subcutaneous tissue of WT and Elovl6 KO mice acclimated to either 24°C or 5°C. (G) Western blot showing UCP1 levels in cold-exposed scWAT. For Elovl6 ratios, n = 8 per group BAT 24°C, n = 5 per group BAT 5°C; for gene expression and western blotting, n = 7 mice per group for both tissues. All mice used were C57Bl/6J male 3–4 months of age. All data were compared by two-way ANOVA analysis. Pairwise comparisons were performed if two-way ANOVA analysis was significant for genotype by t test with Bonferroni correction for multiple comparisons. Error bars ± SEM. ^∗^p < 0.05. Linked to Figure S1.

Next, we investigated the response of Elovl6 KO mice in response to cold acclimation in terms of their fatty acid composition. Mice lacking Elovl6 showed reduced proportions of C18 fatty acids and increased proportions of C16 fatty acids in BAT (Figure 2A) and scWAT (Figure 2B), with subtler changes in liver and muscle (Figures S1C and S1D). Of the fatty acids analyzed, the largest proportional change was for stearate. The overall effect of the changes in fatty acids was reflected in lower C18:C16 ratios in both BAT and scWAT compared to controls (Figure 2C). SCD ratios in both BAT and scWAT of Elovl6 KO mice followed a similar pattern to the C18:C16 ratios, however, showed a smaller magnitude of change (Figure 2D). Importantly, the increase in the C18:C16 ratio caused by cold acclimation was almost entirely ablated by deletion of Elovl6.

To determine whether alterations in the lipidome of BAT in response to cold were necessary for thermogenic adaptation, we next investigated the expression of thermogenic markers in BAT and scWAT. In the BAT of Elovl6 KO mice, only Elovl3 was induced under room temperature conditions (Figure 2E), and no thermogenic markers measured were different between WT and KO animals under cold-acclimated conditions (Figure 2E). However, in scWAT, a major anatomical location of the developmentally distinct beige adipocyte (Murano et al., 2009, Timmons et al., 2007), the Elovl6 KO mice exhibited increased expression of *Ucp1*, *Elovl3*, *Deiodinase 2*, *Lcad*, and *Cpt1b* under cold-acclimated conditions (Figure 2F) as well as UCP1 protein levels (Figure 2G), whereas only Elovl3 was upregulated under room-temperature conditions.

**Figure 3:**
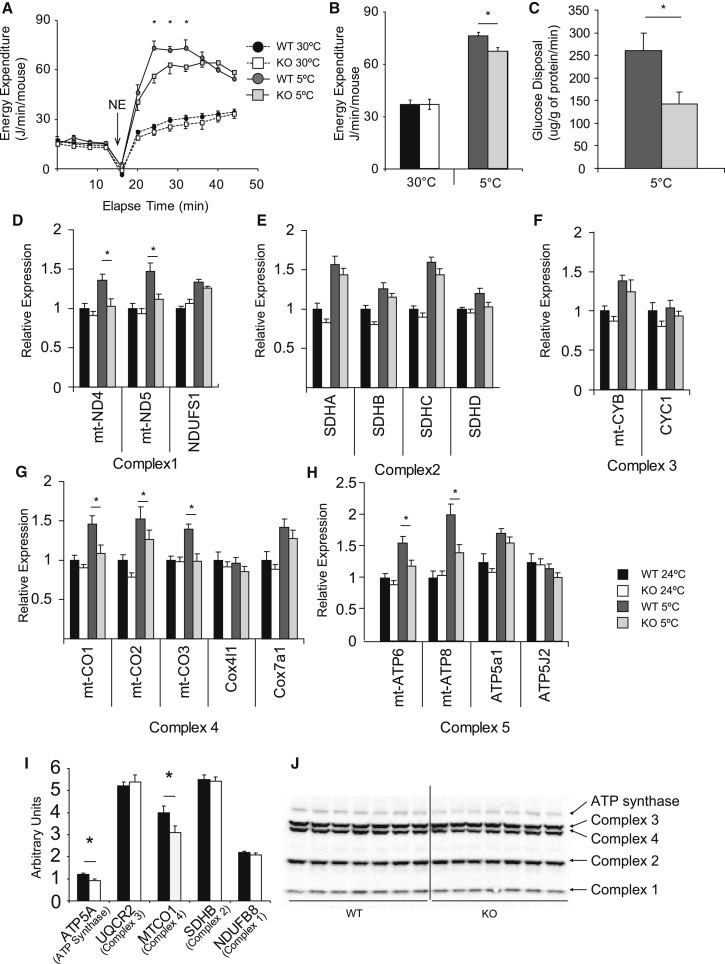
Elovl6 KO Mice Have Impaired Thermogenic Capacity (A) Maximal thermogenic capacity plot showing energy expenditure of WT and Elovl6 KO mice measured after animals were acclimated to indicated temperature. (B) Average of the three highest energy expenditure values observed during maximal thermogenic capacity at each temperature. n = 6 WT and 7 KO mice per group. (C) Norepinepherine-stimulated glucose uptake to BAT; n = 7 WT and 5 KO mice per group. (D–H) Gene expression analysis of the five mitochondrial electron transport chain complexes from WT and Elovl6 KO mice acclimated to either 24°C or 5°C. (D) Complex 1. (E) Complex 2. (F) Complex 3. (G) Complex 4. (H) Complex 5. (I) Quantification of the levels of mitochondrial complex proteins. (J) Western blot showing mitochondrial protein complexes from WT and Elovl6 KO mice, n = 7 mice per group, C57Bl/6J male 3–4 months of age. All data except in (C), (I), and (J) were compared by two-way ANOVA analysis. Pairwise comparisons were performed if two-way ANOVA analysis was significant for genotype by t test with Bonferroni correction for multiple comparisons. Data in (C), (I), and (J) were analyzed by t test. Error bars ± SEM. ^∗^p < 0.05. Linked to Figures S1 and S2.

To directly determine BAT thermogenic capacity, we measured the norepinephrine (NE)-stimulated energy expenditure of WT and Elovl6 KO mice after acclimation to either 4°C or 30°C. The difference between NE-stimulated energy expenditure at these two temperatures has been shown to be UCP1 dependent (Golozoubova et al., 2001). Surprisingly, given the increased expression of thermogenic markers in Elovl6 KO mice, we determined a lower maximal thermogenic capacity in Elovl6 KO mice (Figures 3A and 3B). To confirm this effect, we measured ^14^C 2-deoxyglucose uptake to BAT in response to norepinephrine stimulation in cold-acclimated mice. Mice lacking Elovl6 showed elevated tracer levels in serum for the whole 30-min time course, suggesting lower whole-organism glucose disposal and showed reduced BAT glucose uptake (Figures 3C and S1E–S1G), despite no differences in glucose transporter expression (Figure S1H). In order to determine why mice lacking Elovl6 had reduced thermogenic capacity, we analyzed other genes involved in the thermogenic response in BAT. Analysis of the mitochondrial electron transport chain (ETC) demonstrated that almost all mitochondrially encoded genes were normally expressed at room temperature but failed to show an appropriate induction in Elovl6 KO mice in the cold (Figures 3D–3H). We confirmed on a protein level a reduction in MtCO1 (Figures 3I and 3J).

The reduced expression of the ETC in the BAT of Elovl6 KO mice was consistent with their reduced thermogenic capacity in the cold. Analysis of the lipid composition of mitochondria isolated from the BAT of WT mice demonstrated that they had Elovl6 ratios nearly double the ratio of whole tissue, further supporting a link between ELOVL6 activity and mitochondrial function in BAT (Figure S2A). Furthermore, changes in mitochondrial lipid composition and C18:C16 ratio were much greater in BAT than in liver or muscle (Figures S2B–S2E). Additionally, while changes in SCD ratios in whole tissues were similar to changes in C18:C16 ratios (Figures 2C and 2D), SCD ratios in mitochondria from WT and Elovl6 KO mice were changed less than ELOVL6 ratios (Figure S2F).

In line with the apparent beiging of the scWAT of Elovl6 KO mice in response to cold, we determined that all genes of the mitochondrial ETC were upregulated (Figures S2F–S2J), but not to a greater degree than other BAT markers such as *Ucp1*, suggesting any compensation in scWAT was via recruitment of beige adipocytes.

So far our data suggested that mice lacking Elovl6 had a reduction in BAT thermogenic capacity and a potentially compensatory increase in beige fat located in scWAT. Mice lacking Elovl6 showed no significant differences in body or liver weights at room temperature; however, they had a reduction in WAT mass in the cold (Figures S3A and S3B). To test whether scWAT beiging was compensating for a primary defect in BAT thermogenic capacity, we utilized two paradigms where beige fat activity is minimized—aging to 12 months (12MO) and thermoneutral housing coupled with high-fat feeding (TNHFD).

**Figure 4:**
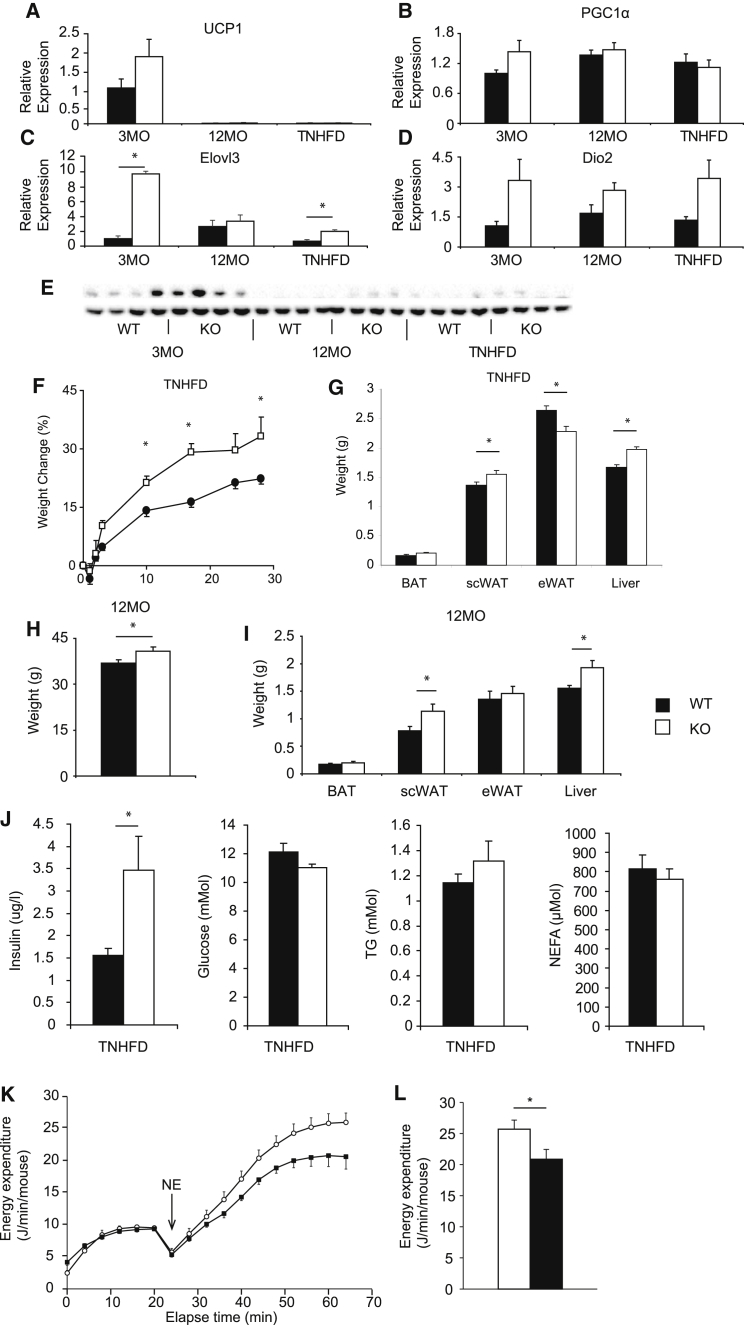
Metabolic Consequences of Disabling Beige Fat in Elovl6 KO Mice (A–E) Gene expression showing the expression of thermogenic markers in inguinal subcutaneous WAT from WT and Elovl6 KO mice housed at room temperature and measured at 3 or 12 months of age or housed at thermoneutrality and fed a high-fat diet from 3 months of age for 4 weeks: (A) *Ucp1*, (B) Pgc1α, (C) *Elovl*3, (D) *Deiodinase* 2, and (E) western blot of UCP1 levels. (F) Percentage weight gain of mice over 4 weeks of high-fat feeding at thermoneutrality. (G) Tissue weights of mice after 4 weeks of thermoneutral housing and high-fat feeding. (H) Body weights of 12-month-old WT and Elovl6 KO mice fed a chow diet and housed at room temperature. (I) Tissue weights of 12-month-old WT and Elovl6 KO mice fed a chow diet and housed at room temperature. (J) Serum biochemistry of fed TNHFD animals. (K) Thermogenic capacity measurements from mice treated with either gambogic acid (1 mg/kg/day) or vehicle. (L) Average of the three highest energy expenditure values observed during maximal thermogenic capacity measurements of GA-treated and vehicle-treated mice. n = 8 per group for 3MO, n = 12 mice per group 12MO, and n = 7 mice per group for TNHFD C57Bl/6J males. All data except in (C), (I), (J), and (L) compared by two-way ANOVA analysis. Gambogic-acid-treated mice were n = 6 per group and were 3-month-old C57Bl/6J males. Pairwise comparisons were performed if two-way ANOVA analysis was significant for genotype by t test with Bonferroni correction for multiple comparisons. Data in (C), (I), (J), and (L) were analyzed by t test. Error bars ± SEM. ^∗^p < 0.05. Linked to Figures S3 and S4.

Both the 12MO and TNHFD mice exhibited greater than 10-fold reductions in *Ucp1* expression in scWAT compared to 3-month-old room-temperature-housed (3MO) controls (Figures 4A and 4E). After TNHFD, Elovl6 KO mice still exhibited significantly higher *Ucp1* expression in ScWAT than wild-types, but their absolute levels of UCP1 expression were greatly reduced compared to 3MO mice. Similarly, the marker of thermogenesis Elovl3 (Figure 4C) was substantially downregulated in both the TNHFD and 12MO groups compared to 3MO controls, whereas expression of *Deiodinase 2* and *Pgc1*α were maintained (Figures 4B and 4D).

To test whether the loss of beige fat activity would result in a metabolic impairment in the Elovl6 KO mice, we measured weight gain and tissue weights in the TNHFD group. In response to TNHFD housing, the Elovl6 KO mice exhibited a 33% weight gain in 28 days compared to only 17% in wild-type mice (Figure 4F). When we analyzed tissue weights, liver and scWAT were significantly heavier in the Elovl6 KO mice; however, the weight of the eWAT depot was decreased (Figure 4G). In response to aging, Elovl6 KO mice were also significantly heavier (Figure 4H) and exhibited increases in scWAT weight and liver weight with no change in eWAT (Figure 4I). Intriguingly, and in line with previous reports (Cohen et al., 2014, Virtue et al., 2012b), the finding that fat mass increases were specific to beige-cell-rich scWAT suggested that a loss of beige fat activity may act locally to facilitate more rapid increase in depot weight. Both TNHFD Elovl6 KO mice and 12MO Elovl6 KO mice exhibited increased weight gain without increased food intake (Figure S3C).

To determine whether a loss of compensatory beige fat activity in the Elovl6 KO mice affected carbohydrate and lipid metabolism, we initially measured serum glucose, insulin, triglycerides, and FFAs in both the aged mice and the TNHFD group. Elovl6 KO mice after TNHFD showed a significant increase in insulin levels compared to wild-type controls (Figure 4J), while 12MO mice only showed a trend (Figure S3D). To determine whether the effects of ELOVL6 on carbohydrate metabolism were secondary to the altered adiposity and liver weights of these mice, we took a further group of animals fed a chow diet and measured glucose tolerance at three temperatures. The Elovl6 KO mice only exhibited impaired glucose tolerance at themoneutrality, suggesting that a loss of sympathetic tone and BAT/beige fat activity may unmask an underlying metabolic defect in the Elovl6 KO mice that is not observed at lower temperatures (Figure S3E).

Recently ELOVL6 activity has been associated to mitochondrial function via negative regulation of the transferrin 1 receptor (TfR1). Mice lacking Elovl6 would be expected to have hyper-active TfR1 signaling. To determine whether activating TfR1 could cause similar changes in BAT to Elovl6 ablation, we treated mice with the TfR1 agonist gambogic acid (GA). GA treatment resulted in reduced maximal thermogenic capacity (Figure 4K) as well as a powerfully inhibiting thermogenic markers and the entire ETC in BAT (Figure S4A) as well as the mitochondrially encoded components of the ETC in scWAT (Figure S4B). Finally, the *Tfr1* receptor was downregulated in the Elovl6 KO mice and downregulated by cold exposure in wild-type mice, consistent with TfR1 signaling having a negative role in BAT thermogenesis (Figure S4C).

## Discussion

Overall, our results define a role for the elongation of non-essential 16 carbon fatty acids to 18 carbon fatty acids in the adaption of BAT to cold. Physiologically, cold acclimation causes an increase in fatty acid chain length in both BAT and scWAT. Ablation of the main enzyme responsible for the interconversion of C16 to C18, Elovl6, reduced overall maximal thermogenic capacity and led to compensatory beiging of white adipose tissue (WAT) depots. Strikingly, in physiological states where beiging of WAT is prevented Elovl6 KO mice exhibit an impaired metabolic profile.

Mice lacking Elovl6 had lower BAT thermogenic capacity, which was associated with a reduction in expression on both an mRNA and a protein level of components of the mitochondrial electron transport chain. Despite these defects in canonical BAT function, and in accordance with previous reports (Matsuzaka et al., 2007), mice lacking Elovl6 were leaner than wild-type controls under RT conditions. The observation that mice lacking BAT function exhibit a lean phenotype at sub-thermoneutral temperatures is consistent with the observations from the *Ucp1* KO mouse (Enerbäck et al., 1997, Liu et al., 2003), which has no functional BAT. *Ucp1* KO mice only manifest an obese phenotype when housed at thermoneutrality, and their obese phenotype is exacerbated by high-fat feeding, due to a loss of diet-induced thermogenesis (Feldmann et al., 2009). In accordance with this concept, mice lacking Elovl6 exhibited obesity when housed at themoneutrality and fed a high-fat diet. Importantly, while high-fat diet stimulates the recruitment and activation of brown adipocytes in canonical BAT, it suppresses beiging of WAT, making this paradigm a method to specifically address defects in BAT rather than beige fat. Furthermore, by housing mice lacking Elovl6 at thermoneutrality it was possible to investigate the metabolic role in terms of carbohydrate metabolism of ELOVL6 independently of effects on BAT.

Mechanistically, ELOVL6 could potentially regulate mitochondrial function through two separate routes. First, mitochondria possess a greater C18:C16 ratio than the rest of BAT, and this ratio is strongly reduced in Elovl6 KO mice. Which specific phospholipid classes within mitochondria are most affected by loss of ELOVL6 remains to be resolved; however, at least one study has shown a broad distribution of stearate and oleate between phosphatidylethanolamine (PE), phosphatidycholine (PC), and Cardiolipin, the three major mitochondrial phospholipids (Ruggiero et al., 1989). An alternative regulatory mechanism is that mitochondrial function is regulated via the TfR1 receptor (Senyilmaz et al., 2015). Stearate has recently been shown to be a negative regulator of the TfR1 receptor, which when activated, impairs mitochondrial function in mammalian cell culture systems. In this study, we demonstrate that activating TfR1 directly impairs thermogenic capacity and reduces markers of thermogenesis and the mitochondrial ETC in BAT and scWAT. Intriguingly, the members of the ETC reduced in scWAT were predominantly mitochondrially encoded genes, similar to those reduced in Elovl6 KO BAT. Additional work will be needed to elucidate the relative balance of changes in mitochondrial fatty acid composition and the TfR1-ELOVL6 signaling pathway.

Although our principal focus is the role of ELOVL6 in brown and beige adipocytes, two previous publications (Matsuzaka et al., 2007, Moon et al., 2014) have investigated the metabolic disease phenotype of Elovl6 KO mice. Matsuzaka et al. (2007) and Moon et al. (2001) found subtly differing impacts of an ablation of Elovl6 on carbohydrate and lipid metabolism. For example, Matsuzaka et al. (2007) did not detect significant differences in steatosis in their model, whereas Moon et al. detected increased liver triglyceride levels (Moon et al., 2001). It was notable that we detected elevated liver weights when *Elovl6* KO mice were housed at thermoneutrality and fed a high-fat diet or were aged, suggestive of increased hepatic lipid content. Furthermore, we found that responses in terms of insulin sensitivity were largely similar to the data of Moon et al. (2001), with no detectable differences in insulin sensitivity at room temperature on a chow diet (data not shown). Where our study differs from either of these reports is that after thermoneutral high-fat feeding we detect significant elevations in serum insulin. Furthermore, in animals fed a chow diet and housed at 30°C we observed impairments in glucose tolerance that were independent from the effects of the body weight changes. It is important to note that each degree reduction in environmental housing temperature that a mouse is exposed to results in a 6% increase in energy expenditure (EE) (Virtue et al., 2012a). This increase in energy expenditure can have dramatic effects on carbohydrate and lipid metabolism and is perhaps the most convincing explanation for the discrepancy between the two previous reports and our own on *Elovl6* KO mouse carbohydrate and lipid metabolism.

Overall, our results demonstrate the necessity for ELOVL6-mediated fatty acid elongation for appropriate mitochondrial function in BAT. We demonstrate on a whole organism level that fatty acid elongation regulates mitochondrial function in vivo. Furthermore, we demonstrate that while beiging of white fat can compensate under “standard laboratory conditions” for defective BAT, a defect in brown fat can be detected by either maximally activating the thermogenesis of an animal after cold adaptation, or by studying the mice under conditions such as thermoneutrality or aging where beiging of white fat is strongly disabled. It is important to note, that thermoneutral high-fat feeding represents the closest analog to the human metabolic state, suggesting that ELOVL6 may play a role in regulating human energy balance.

## Experimental Procedures

### Mouse Generation

Mice heterozygous for a deletion in Elovl6 were phenotyped on a C56Bl6/J background. Mice homozygous for a deletion in Elovl6 (Elovl6^−/−^) and their wild-type littermates were generated by mating heterozygous mice. Gambogic acid (Tocris Bioscience) was dissolved in 20% DMSO, 30% ethanol, and 50% PBS and was administered to mice at 1 mg/kg/day i.p.

All animal breeding and experiments were approved by the UK Home Office and the University of Cambridge. Animals were housed with 12-hr-light and 12-hr-dark cycles. Unless otherwise stated, all animals were studied under fed conditions and at 24°C.

### Diets

Mice were fed either standard breeder’s chow or a 45% fat diet (HFD) (Research Diets D12451); all diets were provided ab libitum.

### Glucose Tolerance Test

Mice were fasted overnight from 4 p.m. until 9 a.m. the next day. Glucose was administered at 2 g/kg i.p. A fixed dose of glucose was given to all mice in the study group based on the average weight of the group.

### Maximum Thermogenic Capacity

Maximum thermogenic capacity of mice was assessed by indirect calorimetry before and after sub-cutaneous norepinepherine injection (1 mg/kg) under pentobarbital (60 mg/kg) anesthesia. Mice were measured at 30°C regardless of previous housing temperature. See the Supplemental Information for further details.

### Norepinepherine-Stimulated Glucose Uptake

Mice were anaesthetized with 60 mg/kg sodium pentobarbital. Mice were injected with 0.2 MBq of [2-14C]-Deoxyglucose ([2-14]DG) (PerkinElmer) i.v. and 1 mg/kg norepinepherine subcutaneously. Blood samples (30 μl) were collected at 10 and 20 min from tail vein for measurement of serum disintegrations per minute (dpm) and blood glucose levels. At 30 min, animals were exsanguinated by cardiac puncture. Tissues were collected and frozen on dry ice. [2-14]DG uptake into tissues was determined by the ZnSO4 BaOH2 precipitation method and normalized to the specific activity of [2-14C]DG in blood to calculate glucose disposal. See the Supplemental Experimental Procedures for further details.

### Lipid Extraction, Derivatization, and FAME Analysis

Lipids were extracted by the Folch extraction procedure. Lipids were esterified to form methyl esters. Methyl esters were analyzed by gas-chromatography flame ionization detection. The identity of species was determined by retention time and compared to a food industry fame standard. Lipid species were quantified based on integrated peak area. See the Supplemental Experimental Procedures for further details.

### Serum Biochemistry

Triglycerides were measured on the Dimension RXL analyzer (Siemens Healthcare). Free fatty acids were measured using the Roche Free Fatty Acid Kit (half-micro test) (kit code 11383175001). Insulin was measured using electrochemical luminescence immunoassay on the MesoScale Discovery immunoassay platform.

### RNA Extraction and Real-Time PCR

RNA was extracted using STAT-60 (AMS Biotech) according to manufacturer’s procedures. Reverse transcription was performed using Reverse Transcriptase System (Promega) according to manufacturer’s instructions. Real-time PCR was carried out using TaqMan or Sybr Green reagents using an Abi 7900 real-time PCR machine using default thermal cycler conditions. Primers are available on request. See the Supplemental Information for further details.

### Western Blotting

Protein was extracted from BAT using RIPA buffer and quantified by the Bio-Rad DC protein assay. 10 μg of protein was loaded per well and subjected to SDS-PAGE in a 4%–12% gradient gel using the Novex NuPage midi system (Life Technologies) and transferred using the iBlot transfer system and reagents (Life Technologies). Membranes were probed for mitochondrial proteins using MitoProfile Total OXPHOS Rodent WB Antibody Cocktail (MitoSciences MS604), UCP1 (Abcam 10983), SREBP1 (Abcam ab3259), and GAPDH (Abcam ab9484).

### Statistics

All statistics were performed using SPSS 18.0. Comparisons between two groups, paired t test, and comparison between two factors two-way ANOVA followed by post hoc testing (Bonferroni correction) were conducted. Data points were excluded if they exhibit a value of more than two SDs from the mean. For all metabolic tests, animals were randomly ordered into metabolic chambers (maximal thermogenic capacity) or to order in which experiments were conducted (GTT, NE-stimulated 2DG uptake). Statistical significance was set at p value <0.05. Specific tests are detailed in the figure legends.

## Author Contributions

S.V. designed and conducted experiments and wrote the paper. C.Y.T. designed and conducted experiments. M.D., R.H., and G.B. conducted experiments. J.L.G. designed and conducted experiments. A.V.-P. designed experiments and wrote the paper.

## Supplemental Information

Document S1. Supplemental Experimental Procedures and Figures S1–S4

Document S2. Article plus Supplemental Information
