# Supplementary material for: Brown Adipose Tissue Thermogenic Capacity Is Regulated by Elovl6
Source: Cell Rep. 2015 Nov 25;13(10):2039–47. doi: 10.1016/j.celrep.2015.11.004 (PMC4688035; doi:10.1016/j.celrep.2015.11.004)
Supplement: Document S1. Supplemental Experimental Procedures and Figures S1–S4 [file mmc1.pdf]

Cell Reports

Supplemental Information

Brown Adipose Tissue Thermogenic Capacity Is Regulated by Elovl6

Chong Yew Tan, Samuel Virtue, Guillaume Bidault, Martin Dale, Rachel Hagen, Julian L. Griffin, and Antonio Vidal-Puig

Brown adipose tissue thermogenic capacity is regulated by Elovl6.

Chong Yew Tan[1]*, Samuel Virtue[1]*#, Guillaume Bidault[1], Martin Dale[1], Rachel Hagen[1], Julian Griffin[2,3], Antonio Vidal-Puig[1,4]#

[1]University of Cambridge Metabolic Research Laboratories, Wellcome Trust-MRC Institute of Metabolic Science, Addenbrooke's Hospital, Cambridge, CB2 0QQ, United Kingdom.

[2] Medical Research Council Human Nutrition Research, Cambridge, CB1 9NL, United Kingdom

[3] The Department of Biochemistry, Tennis Court Road, Cambridge, CB2 1GA, United Kingdom

*These authors contributed equally to this work.

[4] Wellcome Trust Sanger Institute, Wellcome Trust Genome Campus, Hinxton, Cambridgeshire CB10 1SA

#Corresponding sv234@medschl.cam.ac.uk ; ajv22@medschl.cam.ac.uk

Supplemental 1

A

$$\frac{C18{:}0 + C18{:}1n7 + C18{:}1n9}{C16{:}0 + C16{:}1n7} = \text{C18:C16 Ratio} \qquad \frac{C18{:}1n7 + C18{:}1n9 + C16{:}1n7}{C16{:}0 + C18{:}0} = \text{SCD Ratio}$$

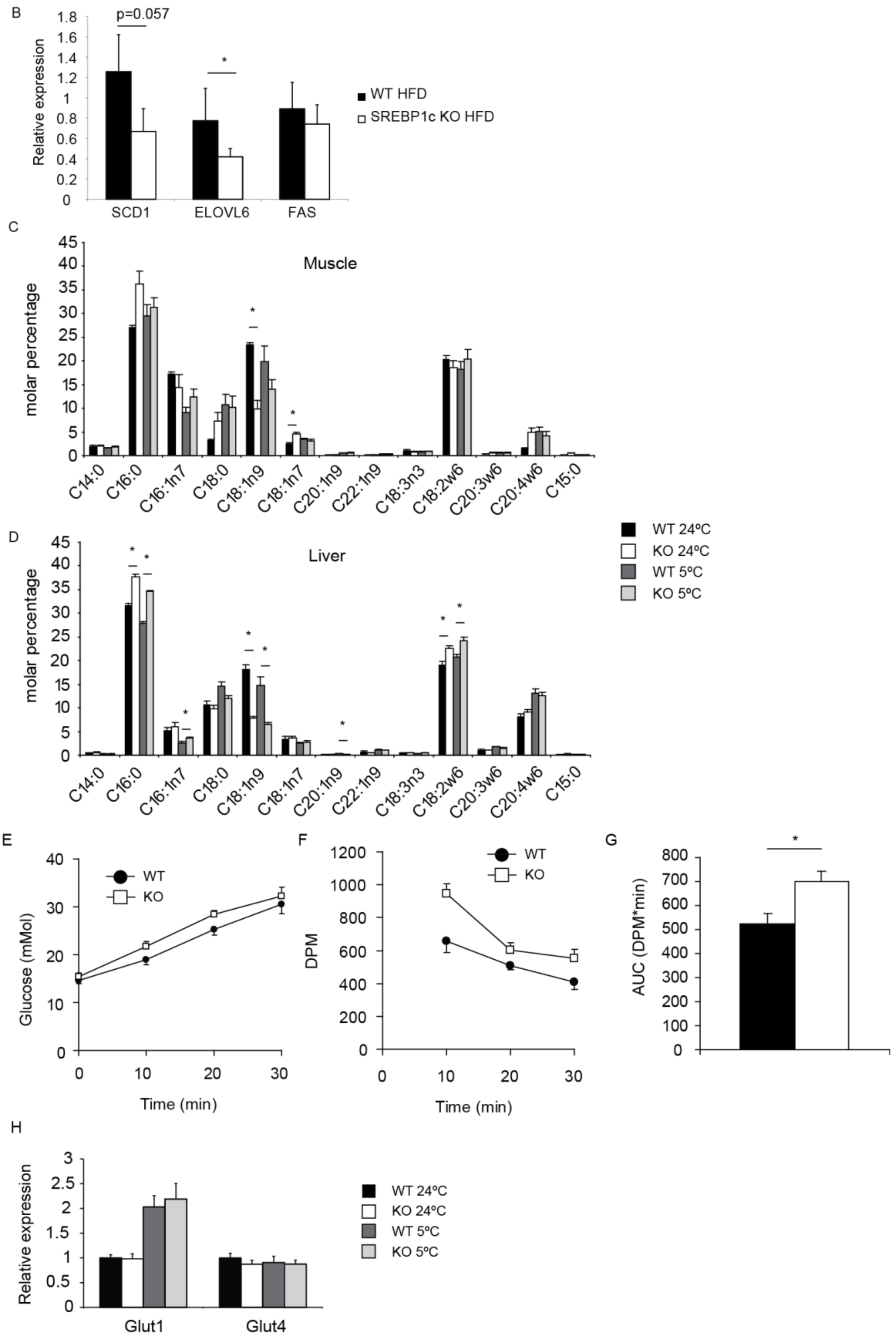

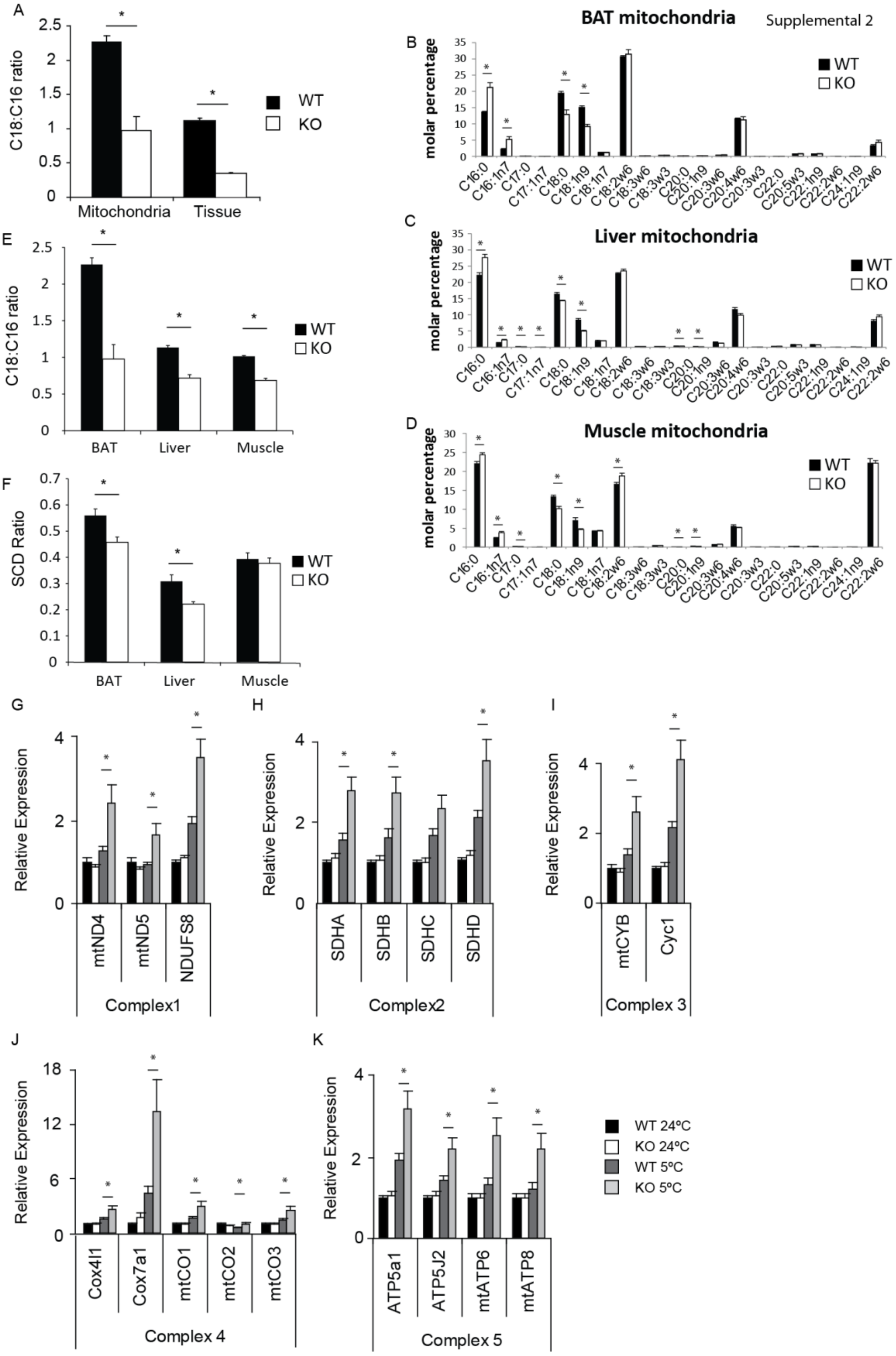
Supplemental 2
A
C18:C16 ratio
Mitochondria
Tissue
WT
KO
BAT mitochondria
B
molar percentage
C16:0
C16:1n7
C17:0
C17:1n7
C18:0
C18:1n9
C18:1n7
C18:2w6
C18:3w6
C18:3w3
C20:0
C20:1n9
C20:3w6
C20:4w6
C20:3w3
C22:0
C20:5w3
C22:1n9
C22:2w6
C24:1n9
C22:2w6
C
Liver mitochondria
D
Muscle mitochondria
E
BAT
Liver
Muscle
F
SCD Ratio
G
Relative Expression
mtND4
mtND5
NDUFS8
Complex1
H
SDHA
SDHB
SDHC
SDHD
Complex2
I
mtCYB
Cyc1
Complex 3
J
Cox4l1
Cox7a1
mtCO1
mtCO2
mtCO3
Complex 4
K
ATP5a1
ATP5J2
mtATP6
mtATP8
Complex 5
WT 24ºC
KO 24ºC
WT 5ºC
KO 5ºC

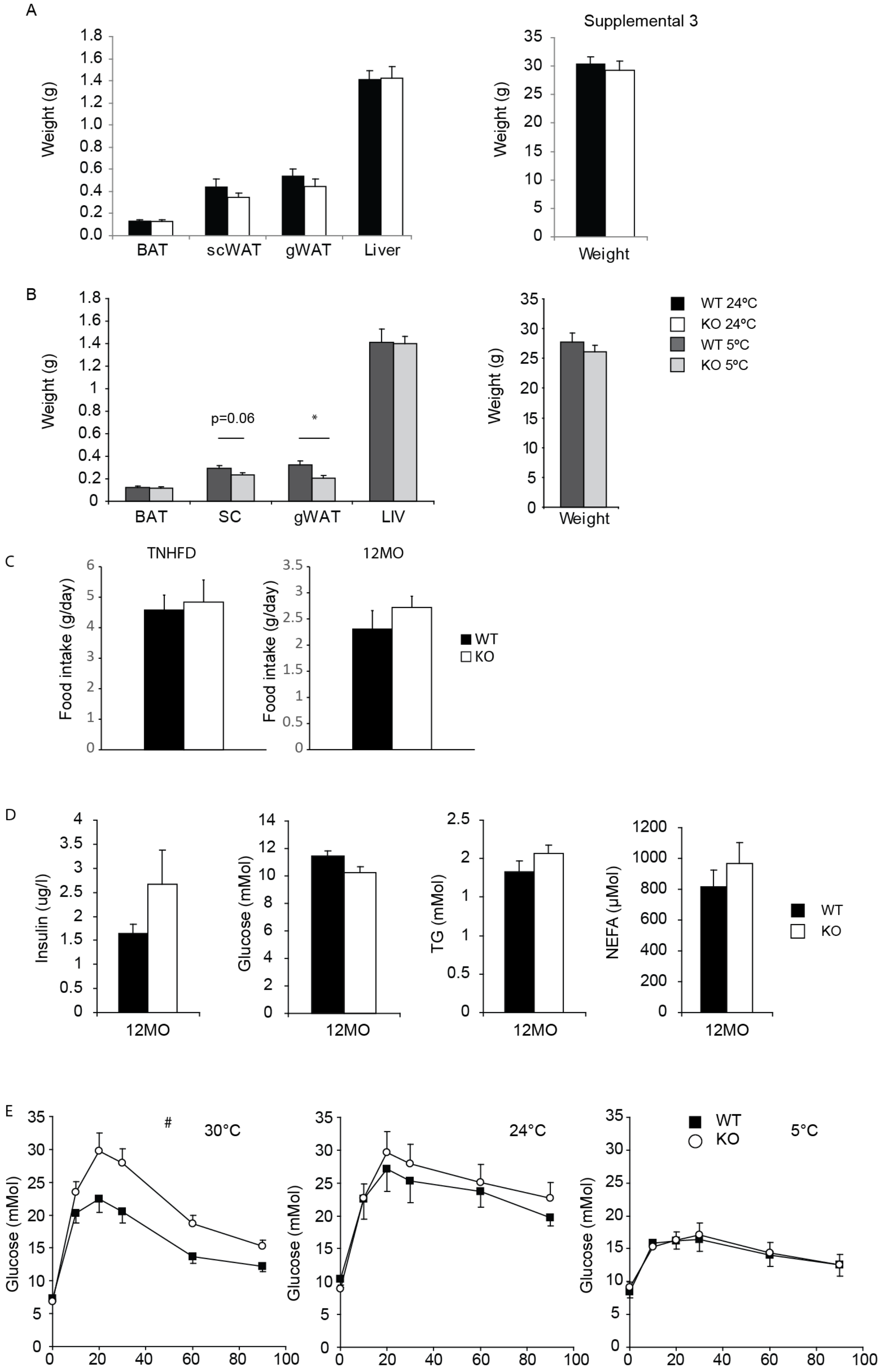
Supplemental 3
A
Weight (g)
BAT
scWAT
gWAT
Liver
Weight
B
p=0.06
*
SC
LIV
WT 24ºC
KO 24ºC
WT 5ºC
KO 5ºC
C
TNHFD
12MO
Food intake (g/day)
WT
KO
D
Insulin (ug/l)
Glucose (mMol)
TG (mMol)
NEFA (µMol)
E
#
30°C
24°C
5°C

Supplemental 4

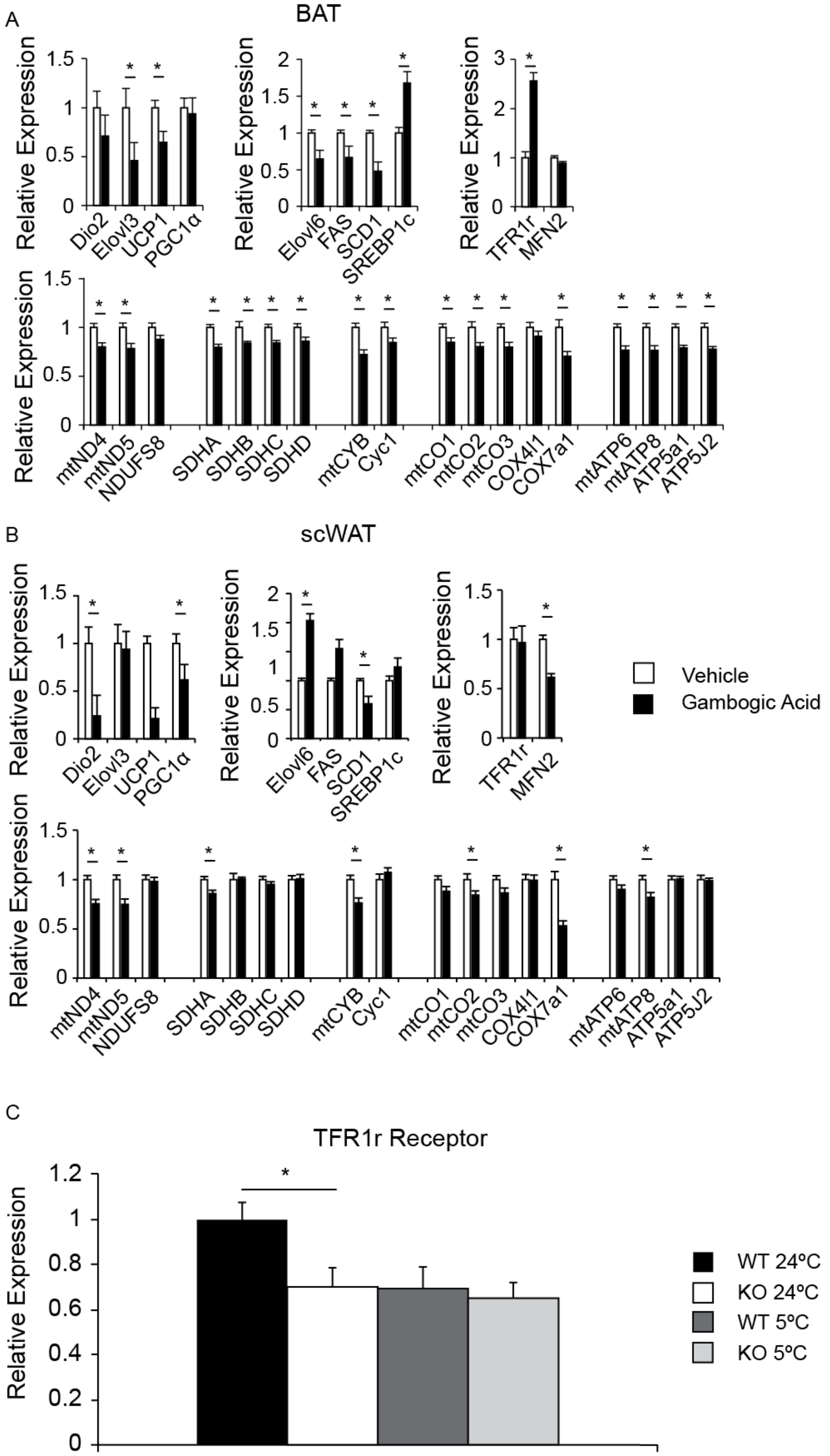

Supplemental information

Supplemental Figure Legends

Figure S1 related to figure 1,2 and 3. A) C18:C16 ratio equation and SCD ratio equation. B) Expression of *Scd1*, *Elovl6* and *Fas* in the brown adipose tissue of WT or *SREBP1c* KO mice on a C57bl/6 background fed a 45% HFD for 4 months from weaning. N=4 per group, male mice. Fatty acid methyl ester analysis of all commonly detected fatty acids of tissues from wild-type mice acclimatized to either 24 or 5°C C) Muscle D) Liver. N= 8 per group for liver and 24°C muscle, n = 4 per group for 5°C muscle. C57Bl/6J male 3-4 months of age. All data compared by two-way ANOVA analysis. Pair-wise comparisons performed if two-way ANOVA analysis significant for genotype by t-test with bonferroni correction for multiple comparisons. E) Glucose levels following injection of NE at T = 0 in anaesthetized mice. F) Disintegrations per minute of 14C-DG in mice injected at T =0 with norepinephrine and 0.2 mBq of 14C-DOG G) Area under curve analysis for C showing Elovl6 exhibit significantly higher serum 14C-DG during the NE-DG disposal assay. E) Expression of *Glut1* and *Glut4* in the brown adipose tissue of warm and cold acclimated *Elovl6* KO and WT mice. N= 7 WT and N = 5 KO for NE-DG assay. N=7 WT and 7 KO for gene expression. C57Bl/6J male 3-4 months of age. All two-group comparisons by t-test. Error bars ± S.E.M. * P<0.05.

Figure S2 related to figure 3 A) Brown adipose tissue Elovl6 ratio of mitochondria and tissue for *Elovl6* WT and KO mice housed at 5°C. Mitochondrial FFA composition of WT and *Elovl6* KO mice after acclimation to 5oC; B) BAT; C) Liver D) muscle. E) *Elovl6* ratio of mitochondrial FFAs. F) SCD ratio of mitochondrial FFAs, n= 8 and 8 for Elovl6 ratio determination for mitochondria. C57Bl/6J male 3-4 months of age. All groups compared by t-test. Expression in warm and cold acclimated WT and *Elovl6* KO mouse scWAT of electron transport genes for: G) Complex 1 H) Complex 2 I) Complex 3 J) Complex 4 and K) complex 5. N= 7 per group per temperature. C57Bl/6J male 3-4 months of age. Data compared by two-way ANOVA analysis. Pair-wise comparisons performed if two-way ANOVA analysis significant for genotype by t-test with bonferroni correction for multiple comparisons. Error bars ± S.E.M. * P<0.05.

Figure S3 related to figure 4 Tissue and body weights of; A) room temperature acclimated *Elovl6* KO mice and B) cold acclimated *Elovl6* KO mice, N=8 per group. All groups compared by t-test. Error bars ± S.E.M. * P<0.05. C) Food intake of thermoneutral-housed high fat fed mice for (N=6 per group) and food intake of 12 month old Elovl6 KO and WT mice (n=6 per group). D) Serum biochemistry of 12 month old Elovl6 KO mice housed at room temperature (N=12 per group). E) Glucose tolerance tests of WT and *Elovl6* KO exposed to either 5, 24 or 30°C for 24 hours prior to and during a glucose tolerance test. Mice were acclimated to 5°C before conducting glucose tolerance tests. N= 8 per group. # P<0.05 for AUC corrected for baseline. Error bars ± S.E.M. * P<0.05.

Figure S4 related to figure 4 Gene expression from A) BAT and B scWAT from mice treated with Gambogic Acid (GA) 1 mg/kg/day or vehicle for 3 weeks. C57Bl/6 male mice housed at 24°C, 3 months old. n=8. C) Expression of the transferrin 1 receptor in brown adipose tissue of warm and cold acclimated WT and Elovl6 KO mice. N=7 WT and 7 KO. C57Bl/6J male 3-4 months of age. Error bars ± S.E.M. * P<0.05.

Supplemental Methods

Mouse generation

Mice heterozygous for a deletion in *Elovl6* (Elovl6 +/-) on a mixed background (C57BL/6 and SV129) were acquired from the European Mouse Mutant Archive (EMMA). Mice on a pure C57BL/6 background were generated by backcrossing mice on a mixed background with wild-type C57BL/6 and assessed by Marker Assisted Accelerated Backcrossing (MAX BAX, Charles Rivers). Mice homozygous for a deletion in Elovl6 (Elovl6 -/-) and their wild-type littermates were generated by mating heterozygous mice. All animal breeding and experiments were approved by the UK Home Office and the University of Cambridge. Animals were housed in a specific pathogen free facility with 12 hour light and 12 hour dark cycles. The light and dark cycles started at 0600 hour and 1800 hour respectively. Unless otherwise stated all animals were studied under fed conditions and at 24°C and a humidity of 55%. Animals represent individual mice from multiple litters, more than one mouse of a given genotype may have come from a given litter in an experimental group

Genotyping

Heterozygous founder mice for a deletion of *Elovl6* (*Elovl6* +/-) were generated through gene trapping performed by lexicon genetics (Elovl6$^{Gt(OST222498)Lex}$). The integration site of the targeting cassette was between exon 2 and 3 of Elovl6 on Chromosome 3. Three primers were used for genotyping.

1) LEXKO 129-5´; 5'-AGGCCAGAGGTATTGAA-TCACC-3´,

2)LEXKO 129-3´; 5'-GACATCATTACTCACTCCAGCC-3´,

3)LTRrev; 5´-ATAAACCCTCTTGCAGTTGCATC-3´.

The expected PCR products were; wild-type, single band at 393 bp, knockouts single band at 138 bp.

Tissue collection

Blood was collected in 1.1ml serum gel separating tubes (Sarstedt 411378005) following cervical neck dislocation and cardiac puncture. The samples were spun at 4°C and snap-frozen on dry ice. Inter-scapular brown adipose tissue (IBAT), inguinal sub-cutaneous white adipose tissue (SC), epididymal (white adipose tissue (gWAT), skeletal muscle (both soleus and gastrocnemius) (SKM), and liver (LIV) were collected, weighed, snap frozen on dry ice and stored in -80°C until used.

Glucose tolerance test

Mice were fasted overnight from 4 pm until 9 am the next day. Blood was collected by tail vein venesection. Blood glucose was measured using the alphaTrak (Abbot) glucosometer calibrated for mouse. Glucose was measured at baseline and at 10, 20, 30, 60 and 90 minutes following intra-peritoneal injection of 2 g/kg of glucose. A fixed dose of glucose was given to all mice in the study group based on the average weight of the group.

Maximum thermogenic capacity

Maximum thermogenic capacity of mice was assessed by indirect calorimetry following sub-cutaneous noradrenaline injection under anesthesia. Indirect calorimetry was performed in an oxymax calorimetry chamber (Columbus, Ohio) which had a 2.7 litre capacity. The Oxymax chamber was housed within a larger temperature controlled cabinet. Room air to the chamber was passed through a heat exchanger to warm it to 30°C. Temperature within calorimetric chambers was continuously monitored and fixed at 30°C. Oxygen consumption and carbon dioxide

production were measured using a custom built oxygen and carbon dioxide monitoring system built by P. Murgatroyd. Airflow rates were set at 400 ml/min. Measurements of oxygen concentration and carbon dioxide concentration in room air and air leaving each cage were measured every 4 minutes. Mice were placed in the calorimetric chamber following anesthesia by intra-peritoneal injection of sodium pentobarbital (60 mg/kg). Baseline gas exchange was recorded once steady state was achieved (at least 3 consecutive stable measurements). Mice were then injected with 1 mg/kg noradrenaline bistartrate (Sigma, UK) sub-cutaneously and returned to the calorimetry chamber. Maximal oxygen consumption rates were typically achieved within 12-16 minutes post injection and were defined either as 3 stable consecutive reading or when oxygen consumption rates began to fall. Energy expenditure was calculated from VO2 and VCO2 using the modified Weir equation.

Norepinepherine stimulated glucose uptake.

Mice were anaesthetised with 60 mg/kg sodium pentobarbital. After 10-15 minutes to allow full sedation the tail vein was cannulated. A basal glucose sample was taken and mice were injected with 0.2 MBq of [2-14C]-Deoxyglucose ([2-14]DG) (Perkin Elmer) IV and 1 mg/kg norepinepherine bistartrate subcutaneously. Glucose was measured at 10, 20 and 30 minutes using an AlphaTRAK (Abbott) glucose meter calibrated for rodents. Blood samples (30 ul) were collected at 10 and 20 minutes from tail vein for measurement of serum DPM. At 30 minutes animals were exsanguinated by cardiac puncture. Tissues were collected and frozen on dry ice. [2-14]DG uptake into tissues was determined by homogenising the tissue in 0.5% perchloric acid followed by centrifugation to deproteinise the samples. Perchloric acid was neutralised using potassium hydroxide and buffered to pH 7 using 50 mM potassium phosphate. An aliquot of the neutralised extract containing both [2-14]DG and p[2-14]DG was analysed by scintillation counting. A second aliquot of the neutralised tissue underwent precipitation by addition of 0.2 volumes of 0.3 N Ba(OH)2 followed by 0.2 volumes of 0.3 N ZnSO4. The precipitate was centrifuged to pellet p[2-14]DG and the supernatant subjected to scintillation counting. The amount of p[2-14]DG was calculated by subtracting the DPM values for the post-precipitation supernatant from the pre-precipitation supernatant after appropriate volume corrections. The pelleted protein samples were resuspended in 0.5 M NaOH and protein concentrations were determined using the BioRad DC assay. Glucose

uptake into tissues was calculated from the DPMs in serum, the blood glucose concentration and the DPMs of the tissue extract and normalised to protein content.

Measurement of serum biochemistry

Triglycerides were measured on the Dimension RXL analyser (Siemens Healthcare). The assays use reagents and calibrators purchased from Siemens. Free Fatty Acids were measured using the Roche Free Fatty Acid Kit (half-micro test) (kit code 11383175001). The assay was modified to run in MicroTitre plate format. Insulin, was measured using electrochemical luminescence immunoassay on the MesoScale Discovery immunoassay platform. All regents and calibrators were purchased from MesoScale Discovery. Assays were run in duplicate. A minimum of two quality control samples were run in each assay.

RNA preparation and Real-time quantitative PCR

Total RNA was isolated using STAT-60 (AMS biotechnology, CS-111) from tissue samples according to the manufacturer's instructions. Tissue were ground in liquid nitrogen using pestle and mortar. 1 ml of STAT-60 was added to the ground tissue, mixed by vortexing and centrifuged at 13,000 g at room temperature for 5 minutes. The supernatant was transferred to chloroform (Sigma 650471) at a ratio of STAT-60:Chloroform 5:1 (v/v). The sample was mixed by vortexing and centrifuged at 12,000g for 15 minutes at 4ºC. The supernatant was added to isopropanol (Sigma 33539) in a STAT-60:isopropanol ratio of 2:1 (v/v) and centrifuged at 12,000g for 10 minutes at 4ºC to pellet RNA. The pellet was washed with 75% ethanol and allowed to dry until ethanol had completely evaporated (pellet appears glassy), and resuspended in RNAse free water (Promega P119C). RNA concentration was determined using nanodrop spectrophotometer (Thermo Fisher scientific, Delaware USA).

500 ng of RNA was used to generate cDNA according to manufacturer's protocol (Reverse Transcriptase System, Promega). Each RNA sample was resuspended in RNAse free water (Promega P119C) to a final volume of 10 µl. To this was added: 4 µl of M-MLV RT buffer (Promega M531A), 2 µl of 25mM MgCl2 (Promega A351B), 2.5µl nucleotide triphosphate (dNTP, Promega U151B) and 0.5 µl 100 mg/ml of random hexamers (Promega C118A). All tubes are heated to 65°C for 5 minutes and returned to ice. 1 µl of reverse transcriptase (Promega M5101) was added (a RT negative control is

also made) and incubated at 37°C for 1 hour. 2 µl from each sample was pooled together to generate a samples for a titration curve. Each sample was diluted 1:75 with RNAse free water (Promega P119C). A titration curve was made by diluting the pooled sample by 1:20, 1:40, 1:80, 1:160, 1:320 and 1:640. RT PCR was performed using TaqMan or Sybr green (Abi). The default thermal cycler conditions were used for all reactions. All primers were designed on Primer Express v3.0. Primers available on request.

Fatty acid extraction from tissue

Total lipids were extracted from frozen tissue using a modified Folch extraction method. A 1.2 ml mixture of chloroform (Sigma 650471):methanol (Sigma 646377) 2:1 v/v was added to tissues in a 2 ml screw top eppendorf tube. Deuterated tridecanoic acid ($C_{13}D_{25}O_2H$, Cambridge Isotopes DLM1392) in chloroform (10mM) was used as an internal standard. 100 mg of ceramic beads (MP biomedicals 6540-434) are added and tissues homogenised in a MP biomedical Fast-Prep 24 homogeniser at 5 Hz for 2 minutes at room temperature. The samples (excluding the beads) were transferred to fresh 2 ml eppendorf tube and 240 µl of water added. Samples are vortexed for 2 minutes and centrifuged at 16,000 g at room temperature for 20 minutes. 700 µl of the lower lipid fraction was transferred to a 7 ml glass tube (SLS TUB1202). A second extraction is performed by adding 700 µl of chloroform followed by vortexing and centrifugation as above. 900 µl of lower lipid fraction is removed and added to the first 700 µl (total 1500 µl). This was dried under nitrogen stream and subsequently saponified and derivitised into fatty acid methyl esters. With each experiment two blank samples containing no tissue were generated, one with internal standard and the other without.

We used 10-15 mg of brown or white adipose tissue, 20-25 mg of muscle and 15-20 mg of liver. Internal standards was added at a volume of 40 ul for BAT or WAT, 15 for muscle and 25 for liver. We resuspended BAT in 1200 ul of hexane, WAT in 100 ul of hexane, muscle in 200 ul of muscle and liver in 400 ul of liver. We then used a split of 45 for BAT a split of 60 for WAT, a split of 55 for muscle and split of 60 for liver.

Lipid saponification and derivation into methyl esters

750 µl of a mixture of chloroform (Sigma 650471):methanol (Sigma 646377) 1:1 v/v was added to previously dried lipids in 7 ml glass bottles. 125 µl of 10% BF3 in methanol (Sigma 134821) was then added. The bottles were sealed and incubated in an oven at 80°C for 90 minutes. The samples were allowed to cool, and 1 ml of n-Hexane (Sigma 34859) and 500 µl of water (Sigma 34877) were added. The samples were mixed by vortexing and centrifuged at 2500 rpm on a Thermo Scientific Sorvall Legend RT+ centrifuge at room temperature. The upper organic layer was transferred into glass vials and dried under a nitrogen stream.

Gas chromatography of fatty acid methyl esters (FAME)

Gas chromatography was performed on a Thermo Finnigan Focus GC coupled to a FID detector (GC-FID) and an AS3000 auto sampler. A Thermo Scientific TR-FAME column (length: 30 m, inter diameter: 0.25 mm, film size: 0.25 µm, #260M142P) was used with helium as carrier gas. Inlet and FID detector temperature was set at 230°C and 250°C respectively. Dried FAME samples were resuspended in n-Hexane (Sigma 34859) according to the volumes listed in table 2.18. 5 µl of this solution was injected for analysis. The oven programs used depend on the sample of interest; mammalian tissue or human plasma. These are presented in the following table. Samples were run at random to control for within run effects. Identification of FAME peaks was based on retention time and made by comparison with those in external standards (Restek 35077 Food industry FAME mix and Supelco 46904 Vaccenic Methyl ester).

The GC oven program used for all samples involved a carrier gas flow of 1.5 ml/min. The temperature steps used were; Step 1 100°C for 2 minutes, Step 2 25°C per minute to 150°C, Step 3 2.5°C to 162°C followed by a hold time of 3.8 minutes, Step 4 4.5°C per minute to 173°C followed by a hold time of 5 minutes, Step 5 5°C per minute to 210°C, Step 6 40°C to 230°C followed by a hold time of 0.5 minutes.

Identification and quantification of FAME peaks

GCFID .dat files were converted into .raw files for import into Xcalibur 2.0 (release 14 Feb 2005). Expected retention time for individual peaks was compared against that of the external standard. Peak detection was made using Interactive Chemical Integration System *(*ICIS) algorithm and peak areas was used for quantification. Background noise for each peak was removed by subtracting the values found in

blank samples. The samples were then normalised to internal standard and expressed as molar% values by dividing each peak by the sum of all the peaks for a given sample.
